# Data supporting the role of electric field and electrode material on the improvement of the ageing effects in hydrogenated amorphous silicon solar cells

**DOI:** 10.1016/j.dib.2015.07.020

**Published:** 2015-07-23

**Authors:** Andrea Scuto, Luca Valenti, Silvio Pierro, Marina Foti, Cosimo Gerardi, Anna Battaglia, Salvatore Lombardo

**Affiliations:** a CNR IMM, VIII Strada, 5, Z.I., 95121 Catania, Italy; b DIMES, Università della Calabria, Via P. Bucci, 46, 87036 Rende, Italy; c STMicroelectronics, Stradale Primosole, 50, 95121 Catania, Italy; d 3SUN S.r.l., Contrada Blocco Torrazze sn - Z.I., 95121 Catania, Italy

**Keywords:** Hydrogenated amorphous silicon, a-Si:H, Solar cell, Mitigation of ageing effects, Reverse bias stress

## Abstract

Hydrogenated amorphous Si (a­Si:H) solar cells are strongly affected by the well known Staebler–Wronski effect. This is a worsening of solar cell performances under light soaking which results in a substantial loss of cell power conversion efficiency compared to time zero performance. It is believed not to be an extrinsic effect, but rather a basic phenomenon related to the nature of a­Si:H and to the stability and motion of H­related species in the a­Si:H lattice. This work has been designed in support of the research article entitled “Role of electric field and electrode material on the improvement of the ageing effects in hydrogenated amorphous silicon solar cells” in Solar Energy Materials & Solar Cells (Scuto et al. [1]), which discusses an electrical method based on reverse bias stress to improve the solar cell parameters, and in particular the effect of temperature, electric field intensity and illumination level as a function of the stress time. Here we provide a further set of the obtained experimental data results.

**Table:** Specifications table

Subject area	Physics
More specific subject area	Photovoltaics
Type of data	Tables, figures
How data was acquired	Cascade probe station with micro chamber - HP 4156B semiconductor parameter analyzer - 92191–1000 Newport solar simulator - Thermostatic chuck with a Temptronic thermal controller working under N_2_ flux
Data format	Analyzed
Experimental factors	The hydrogenated amorphous Si (a-Si:H) solar cells used in the present study were single-junction p–i–n cells with p and n-type a-Si:H layers of both 20 nm thicknesses and intrinsic (i) a-Si:H layer of various thicknesses. The analyzed samples had a AGC ASAHI GLASS VU-type substrate with ≈700 nm thick SnO_2_:F as transparent conductive oxide (TCO) deposited by sputtering; the cells were deposited by plasma enhanced chemical vapor deposition (PECVD) under the same conditions at 255 °C; the top electrode was a 900 nm thick ZnO:Al (AZO) TCO. The entire solar cell layer sequences was glass substrate/SnO_2_:F/p–i–n a-Si:H/AZO. The final geometries (circular with diameters varying from 0.01 to 0.64 cm) were defined by photolithography and selective etching of the AZO/p–i–n films.
Experimental features	All the solar cell electrical measurements were performed in substrate configuration, i.e. with the illumination light entering from the top AZO contact.
Data source location	Institute for Microelectronics and Microsystems, National Research Council, Catania, Italy
Data accessibility	Data are with this article

Value of the data

• The solar cell improvement under reverse bias stress application is quantitatively reported;
• Data of the temperature dependence of the solar cell parameter change under reverse bias stress are shown;
• Clear evidence of the reversibility of the solar cell parameter change depending on the polarity of the applied stress is shown.

## 1 Data, experimental design, materials and methods

### 1.1 Light induced degradation of solar cells under short circuit condition

**Fig. 1:**
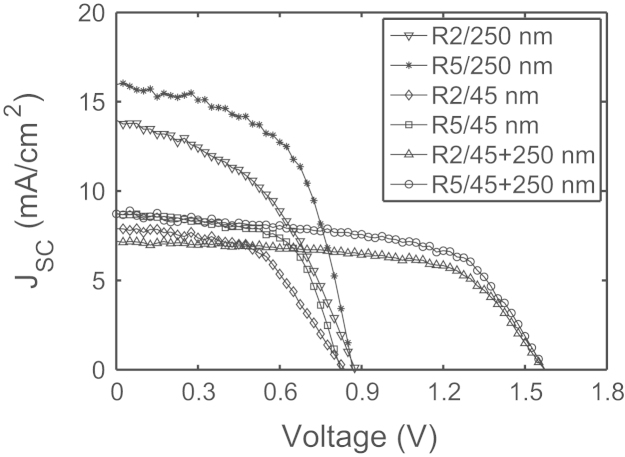
Solar cells comparison showing the differences between the two hydrogen–silane ratios (*R*=2 and *R*=5) combined with the three different intrinsic thickness layers (*i*=45, *i*=250 and *i*=45+250).

To define the sample preparation conditions we have studied the role of the H_2_/SiH_4_ ratio during the PECVD deposition of the a-Si:H layers at 255 °C on the time zero performance of the solar cells, given the important role played by the H_2_ dilution [2], [3], [4], [5], [6]. We prepared two different typologies of samples using various different dilution ratios *R*, defined as the H_2_/SiH_4_ ratio. A number of a-Si:H solar cell types were used in this analysis. One group was single-junction p–i–n cells with p and n-type a-Si:H layers of both 20 nm thickness and with the intrinsic (i) layer of either 45 nm or 250 nm thickness. The second group was a tandem a-Si:H/a-Si:H cell where the two i layers were 45 nm and 250 nm, respectively. Fig. 1 shows the *I*–*V* characteristics of these samples measured under AM1.5G spectrum with illumination intensity of 1.5 suns. From the figure it is evident that in each case the samples with *R*=5 dilution show better short circuit current. The effect is attributed to a better photo-carrier lifetime. In all cases, however, the a-Si:H films are amorphous, not micro-crystalline, and without any clear sign of Si nanocrystals, as shown by Raman and TEM analysis (not reported). For all the experiments reported in the following part of the paper, we have used single junction a-Si:H solar cells with 250 nm *i* layer and prepared with a dilution *R* equal to 5.

**Fig. 2:**
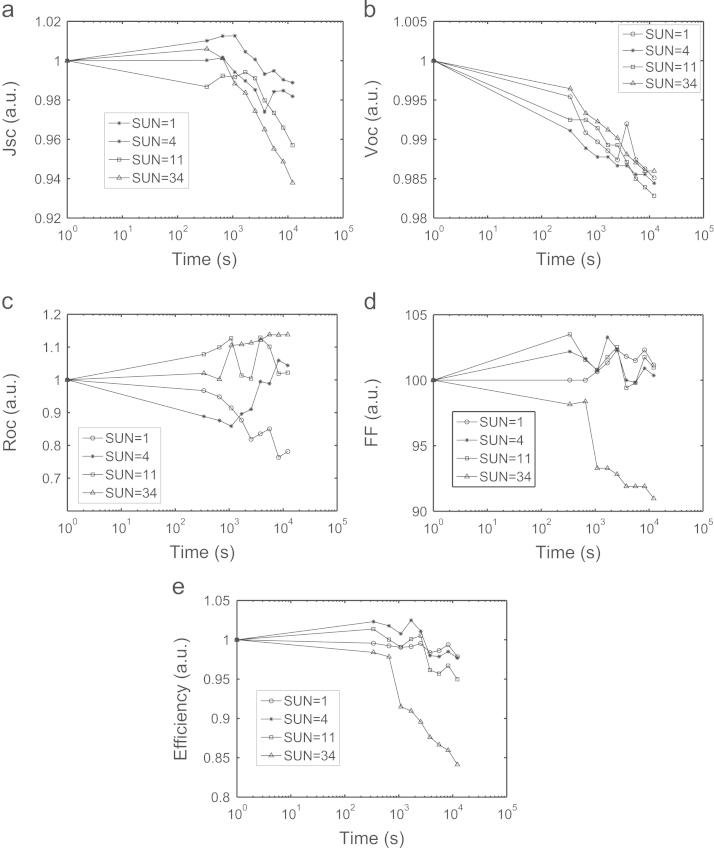
Normalized data trend analysis (short circuit conditions) for (a) *J*_SC_, (b) *V*_OC_, (c) *R*_OC_, (d) FF and (e) efficiency as a function of light soaking time for increasing sun intensity.

To study the degradation of our solar cells under light soaking and, consequently, to define a reference baseline, we have analyzed the effect of light soaking stress under short circuit conditions on all the major solar cell parameters/figures of merit. As expected, under this condition it is observed an increasing solar cell degradation as a function of stress time, and the degradation rate is an increasing function of the incident light intensity [1], [7] (Fig. 2).

## 2 Quantitative evaluation of the solar cell improvement under reverse bias stress

We now show how the application of a strong reverse bias during the light soaking dramatically changes the wear out kinetics. Table 1 reports data of the major solar cell parameters/figures of merit as function of the stress time observed by applying a fixed reverse bias of −12 V under a light exposure with AM1.5G spectrum at 1.5 equivalent suns [1]. By observing the values, it is evident that the solar cell characteristics under the reverse bias stress are improving as the stress time increases.

## 3 Analysis of the effect of temperature during reverse bias stress

**Fig. 3:**
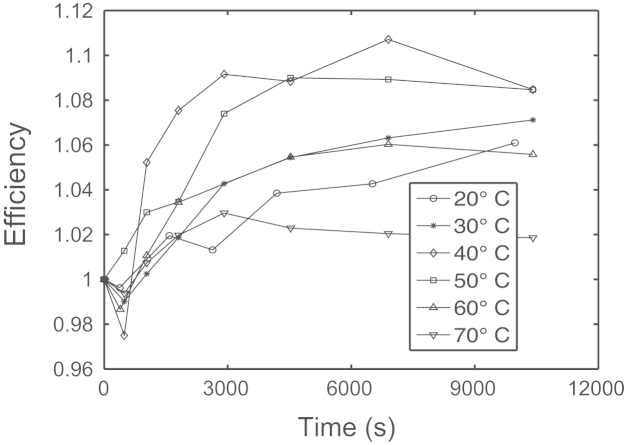
Normalized efficiency data trend analysis as a function of stress time for increasing temperature intensity observed applying a fixed reverse bias of −12 V under a light exposure of 1.5 equivalent suns.

As reported in [1], it was observed that the application of a strong reverse bias stress to the a-Si:H solar cells rather than simply slowing down the wear out rate under light soaking [8], indeed improves the solar cell characteristics. We have therefore analyzed the role of the solar cell temperature on the improvement kinetics in reverse bias stresses at −12 V under a light exposure of 1.5 suns. Fig. 3 shows the effect of the solar cell temperature during the stress. It is evident that the largest solar cell improvement effect is around 40–50 °C, which represents in this case the ideal heating treatment. Lower or higher temperatures produce less improvement. This indicates that the temperature represents a further important factor to be considered in the solar cell recovery/improvement mechanism. This circumstance may be due to the fact that either the solar cell improvement is related to a short range atomic species diffusion phenomenon or other mechanisms become important at larger temperatures.

## 4 Reversibility of the solar cell parameter change depending on the stress polarity

**Fig. 4:**
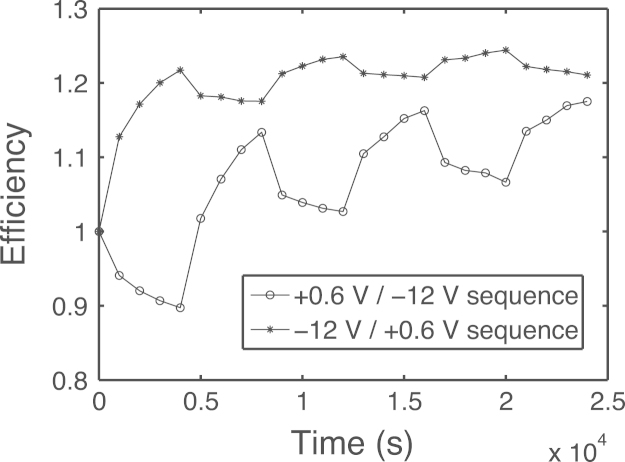
Normalized efficiency data trend analysis as a function of stress time in response to contrary and alternating voltage bias (+0.6 V and −12 V) applied for 4000 s each under a light exposure of 1 equivalent sun.

As observed in the case of p single substrates [1], where the sheet resistance goes up and down following the sign of the applied voltage pulse, also in the case of the complete a-Si:H solar cells we observe reversible changes in the solar cell power conversion efficiency finding monotonic trends in response to forward and reverse bias stress. As example, we report the results of experiments performed with stresses in forward (F) and reverse (R) bias, +0.6 V and −2 V, respectively. Each voltage stress lasted 4000 s and it was performed under a light exposure of 1 equivalent sun. Fig. 4 reports the normalized solar cell power conversion efficiency as a function of time for two different stress sequence conditions, i.e., RFRFRF and the opposite FRFRFR. That is, in one case we start to stress the cell with −12 V (noting a considerable increment of efficiency) and in the other we first apply a positive bias of +0.6 V (noting a fall in efficiency). As clearly shown in Fig. 4, in both cases we observe a noticeable solar cell efficiency growth.

## Figures and Tables

**Table 1:** Quantitative evaluation of the solar cell improvement under reverse bias stress. Major solar cell parameters/figures of merit as a function of stress time at fixed reverse bias of −12 V under a light exposure of 1.5 suns.

**Stress time (s)**	***J***_**SC**_**(mA/cm**^**2**^**)**	***R***_**OC**_**(Ω cm**^**2**^**)**	***R***_**SC**_**(kΩ cm**^**2**^**)**	**FF (%)**	***V***_**OC**_**(V)**	**Eff. (%)**
*t*=3	11.13	11.61	1.69	60.58	0.802	5.39
*t*=35	11.14	11.44	1.59	61.00	0.805	5.45
*t*=65	11.16	11.41	1.56	61.53	0.805	5.50
*t*=100	11.15	11.33	1.92	61.78	0.806	5.52
*t*=400	11.15	10.48	1.66	63.02	0.810	5.67
*t*=1000	11.18	10.84	1.62	61.47	0.822	5.62
*t*=5000	11.21	10.27	1.82	64.52	0.835	6.03
*t*=8000	11.24	8.24	2.28	66.48	0.827	6.16
*t*=11,000	11.28	8.26	1.92	66.00	0.835	6.17
